# Total Knee Arthroplasty in a Knee Locked in Extension: A Case Report

**DOI:** 10.7759/cureus.78443

**Published:** 2025-02-03

**Authors:** Akhilesh Pradhan, Najibullah Ghasemi, Howard Tanner, Pierre Nasr

**Affiliations:** 1 Orthopaedics and Traumatology, Northampton General Hospital NHS Trust, Northampton, GBR

**Keywords:** extension deformity, knee arthroplasty, limb deformity, musculoskeletal biomechanics, orthopaedic surgery

## Abstract

A native arthritic knee locked in full extension is a rare presentation to an orthopaedic clinic. Often, fixed deformity cases present with an element of fixed flexion due to either a mechanical or non-mechanical block, but a full extension deformity has not been reported in the literature. The treatment of these complex cases often involves consideration of the biopsychosocial impact of the deformity, and holistic patient care is warranted.

This case report explores the symptoms, investigations, management, and postoperative recovery of a 60-year-old patient presenting with a rare fixed extension deformity. The condition had a significant impact on the patient's physical and psychological state, and its impact on quality of life should not be dismissed. Multiple imaging modalities and surgical options were considered prior to surgical management with total knee arthroplasty.

The management of this condition was demonstrated to be suitably treated with total knee arthroplasty with good postoperative function and recovery. However, further larger study series are required to provide more robust evidence as to the optimal management strategy.

## Introduction

A knee locked in extension is a very rare entity presenting to a hip and knee clinic and has not been previously explored in the literature. It is important to emphasise that it presents differently from a traditional locked knee. The traditional locked knee presents a unique orthopaedic emergency that often requires emergent management to ensure optimal patient care but is a more common presentation compared to the case reported in this article [1]. The common definition/current understanding of a "locked" knee often refers to a patient unable to fully extend or fully flex their knee, which results in a position where the knee is flexed to 30-40 degrees. In the setting of the acute orthopaedic take, the patient often presents with a hot, swollen knee with a preceding history of trauma or an event where a "pop" or a "giving way" is experienced [2]. 

The most common causes of this presentation are meniscal injury/tear, ligamentous injury, or loose body/osteochondral fragment [3]. This involves a mechanical block between the femoral condyles and the tibial plateau that prevents the patients from obtaining a full range of motion. Furthermore, it is important to differentiate between these mechanical causes versus non-mechanical causes of knee locking, i.e., pseudo-locking, which may result from muscular imbalance, quadriceps inhibition, pain inhibition, or psychological issues (in the absence of physical/mechanical block) [4].

Despite these relatively uncommon presentations, an atraumatic knee locked fully in extension is a rare entity and has not been explored within the literature. There is a paucity of evidence regarding the causation of this rare subgroup of the locked knee; therefore, the authors cannot provide further evidence from the literature regarding mechanical versus non-mechanical causes in the same context as a knee locked in flexion. This case report highlights the initial presentation, progression of the patient's symptoms, surgical management, and, ultimately, postoperative outcome for a knee locked in full extension with no active or passive range of motion.

## Case presentation

A 60-year-old patient presented to the elective hip and knee clinic at a district general hospital with a knee locked in extension. She did not report any history of trauma, but her symptoms presented suddenly whilst she was pushing a shopping trolley in a supermarket car park and felt instant pain in her right knee with difficulty in weight bearing. She presented with general tenderness over the medial and lateral joint lines and did not have any active or passive knee flexion. She did not have any relevant co-morbidities or social history that would contribute to her condition; however, she did report having issues with intermittent knee locking (limited to 20-30 degrees of flexion) throughout her 20s, which self-resolved with gentle exercises and non-operative management. She did not report any episodes of patellar instability or subluxation, which may have contributed to scarring (and, therefore, restriction in range of motion). Regarding occupation, she had a manual job working as a supermarket assistant, involving long periods of time standing at tills or on the aisles (30-40 years of manual work).

Clinical findings

On inspection, the patient presented in a wheelchair with leg support and her right knee locked in full extension. She was unable to mobilise out of her wheelchair and did not have sufficient balance in using her crutches. There was no obvious joint effusion, and the patella was centred appropriately over the knee. There were no obvious scars, erythema, or skin changes. On palpation, she presented with generalised medial and lateral joint line tenderness but no point tenderness over any region. The active and passive range of motion was fixed entirely at zero degrees of extension with no hyperextension and no ability to actively or passively flex the knee. Ligament testing in full extension did not demonstrate any obvious laxity but was understandably difficult to examine.

Diagnostic assessment

Radiographic imaging demonstrated bicompartmental arthritic wear with a larger lateral compartment wear profile and approximately 10 degrees of valgus alignment. Upon calculation of the Insall-Salvati ratio, patella alta was noted (ratio of 1.3). The authors would like to emphasise that the ratio stated here is an approximation; a true Insall-Salvati ratio relies on a lateral radiograph with 30 degrees of flexion, which was not possible in this instance due to the knee being held in extension by the patient. The role of patella alta, in this case, is unclear but is thought to have contributed to altered biomechanics and perceived stability of the extensor mechanism and may have contributed to the psychological component of this condition, with the patient reporting instability symptoms early in her presentation. It is unlikely that valgus alignment and patella alta resulted in a full extension deformity but are likely to have contributed to the overall presentation. The presence of patella alta did not alter the choice of surgical approach, and regardless of a valgus deformity with patella alta, a medial parapatellar approach was deemed suitable.

**Figure 1:**
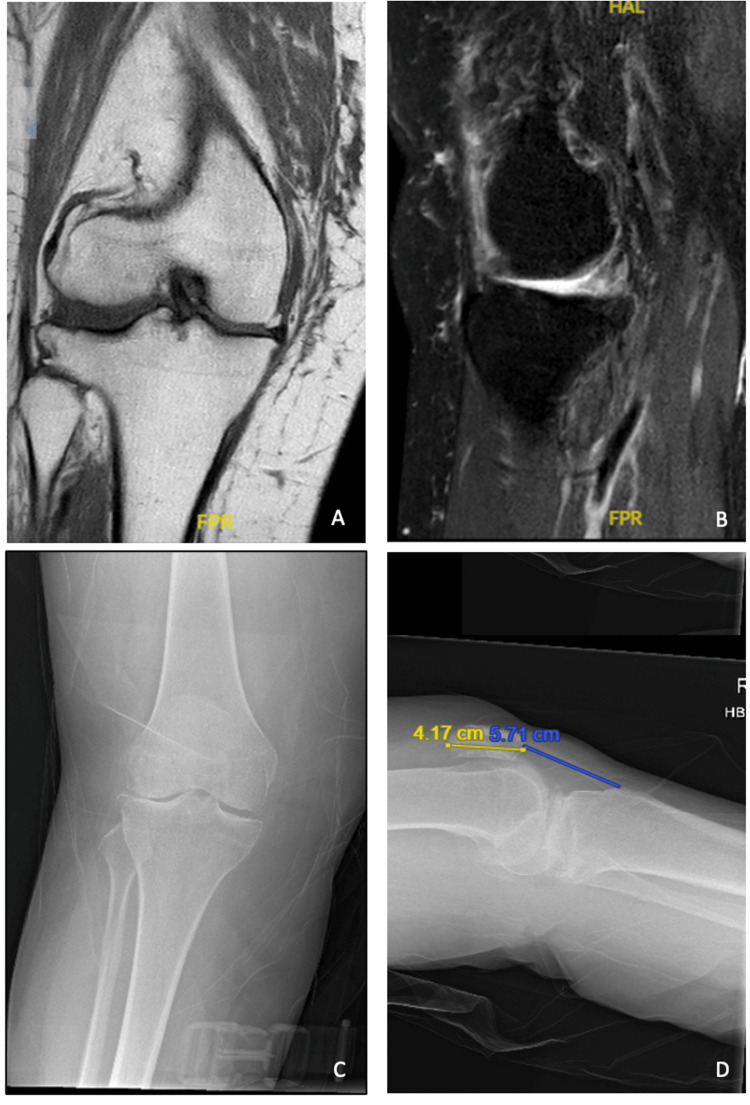
Preoperative MRI and radiographic imaging after initial consultation A, B: Degeneration of medial meniscus and degenerative compartmental changes on preoperative MRI scan; full thickness cartilage loss is visualised with joint space narrowing predominantly in the lateral compartment. C, D: Preoperative radiographs demonstrating generalised degenerative wear profile and patella alta with significant bicompartmental arthritic changes.

Further MRI imaging did not demonstrate any large bucket handle meniscal tears, with congruency of her anterior and posterior cruciate ligaments and no osteochondral loose bodies; however, severe lateral compartment degenerative changes were identified with full-thickness cartilage loss of the lateral compartment (Figure 1). Panels A and B demonstrate MRI findings consistent with lateral compartment degenerative wear with full thickness cartilage loss and degenerative wear of the lateral meniscus with fraying/wear. Panels C and D demonstrate anteroposterior and lateral radiographic imaging demonstrating a 10-degree valgus alignment of the knee with degenerative changes consistent with bicompartmental osteoarthritis. The authors do not predict that the degenerative wear of the meniscus contributed to any mechanical block limiting flexion; however, degenerative joint disease in the lateral compartment may have contributed as a mechanical block to flexion/difficult range of motion. 

As a result of her clinical symptoms and MRI findings [5], she underwent manipulation under anaesthesia and diagnostic arthroscopy with meniscal debridement. Upon induction of general anaesthesia, the loss of muscle tone enabled knee flexion to 90-95 degrees during manipulation, which was a marked improvement from her initial presentation. There was the absence of any mechanical block, no signs of patella subluxation, and no resistance to flexion range; hence, suggestive of a non-mechanical/psychological component of the presentation. Furthermore, knee arthroscopy did not reveal any osteochondral lesion, any significant scarring or inflammation, bucket handle tear, or large incarcerated meniscal fragment in the joint contributing to her symptoms. The degenerative lateral meniscus was debrided to reduce any contributory mechanical block (which the authors did not feel was a major cause of this deformity). Postoperatively, the patient underwent physiotherapy and a short duration of a continuous passive motion (CPM) machine. Unfortunately, within 24 hours of the general anaesthetic, the patient reverted to full extension deformity with no active or passive flexion achieved. The use of manipulation and diagnostic arthroscopy was performed to confirm the role of any physical, mechanical block to range of motion but also to identify the contribution of non-mechanical causes, e.g., psychological distress/ pain inhibition, which may have been present when the patient was conscious. The authors note the varying benefits of CPM machines in the literature, with no definitive evidence of their use in postoperative protocols. The use of CPM is not standard practice within our unit for knee arthroplasty patients but was deemed necessary in this rare case to optimise the range of motion by all means. Ultimately, the role of CPM was limited to providing functional recovery to the patient, and further interventions were required. 

Therapeutic intervention

Subsequently, the patient underwent a period of non-operative management with ongoing physiotherapy assessments and exercise provision, which did not provide any benefit to range of motion or aid in the improvement of flexion range. The authors speculate that failure of non-operative modalities was likely due to the progression of degenerative wear, non-compliance with treatment, and further pain inhibition/psychological factors reducing range of motion. After an interval of one year, the patient re-presented to the elective hip and knee clinic with ongoing concerns due to lack of mobility and its consequent effect on her quality of life. She had been discharged from her job due to being unable to mobilise and being uncomfortable standing for long periods of time. She was mobilising with difficulty using crutches. There was a significant impact during this period on her mental health. She was counselled extensively by the team, and her case was discussed in the local complex case discussion meeting. Discussion with the local network of hip and knee arthroplasty surgeons did not attribute any major cause to the patient's presentation, but a consensus was reached regarding management with elective total knee arthroplasty. Valgus knee alignment in the context of bicompartmental degeneration of the knee joint provided a reasonable indication for total joint replacement. 

The patient was reviewed in the clinic and extensively counselled regarding the aim of surgery, potential risks, and the possibility that surgery would be unable to correct her fixed extension deformity. The patient was keen to undergo total joint arthroplasty and felt that even a minimal improvement in flexion range would have a large impact on her quality of life. Pain due to her knee extension was largely controlled with Zapain®, a morphine derivative, and simple analgesia (paracetamol, ibuprofen). Prior to operative intervention, she underwent an online pain management session with author HT, a session developed by Stanford University to aid patients in managing pain prior to and after their surgery. These sessions are offered to all patients with high preoperative pain scores and low Oxford Knee Scores (<20/48). Pain management is an important area of current interest and has been named by the James Lind Alliance as one of the top priorities for further management solutions. Whilst our unit has protocols for postoperative analgesia, there are no protocols for preoperative analgesia administration. Arthroplasty patients may benefit from neuropathic pain medications in addition to medications addressing nociceptive pain; however, this is an area that requires further research and evidence-based management, especially in complex cases where patients have additional requirements/needs. 

Total knee arthroplasty was conducted using the DePuy Synthes Sigma™ PFC® primary knee system. Patient positioning was technically challenging due to her extension deformity. It was noted that during spinal anaesthesia, the lack of muscle tone did not enable a better flexion range of motion during her second episode of manipulation under anaesthesia. This resulted in a difficult exposure, with the incision and initial dissection having to be conducted in full extension. Gradually, with soft tissue releases, the knee was able to be flexed. The use of two foot rolls (one placed at 20-30 degrees and another placed at 80-90 degrees) was essential during positioning and enabled stabilisation of the limb as the soft tissue releases enabled further flexion. The medial para-patellar approach with subsequent eversion of the patella tendon enabled a greater degree of flexion, enabling further soft tissue releases (principally of adhesions and synovial tissue) that allowed further flexion. 

Intraoperatively, there was no significant scarring of the soft tissues or the ligaments. However, there were significant osteophytes at the posterior aspect of the joint, which were fully excised. Grade IV wear was present in both medial and lateral compartments, with a thin patella and a hypoplastic lateral femoral condyle. The posterior cruciate ligament was preserved, and a cruciate retaining implant was placed with knee stability achieved using gap balancing methods. Minimal soft tissue releases were conducted to prevent over-loosening of the joint spaces.

Despite adequate bone cuts to the femur and tibia, there were concerns about implant "lift-off" during the trial and, therefore, adjustments were made to increase the flexion gap (including downsizing of the femoral component, anterior referencing, and increasing posterior tibial slope) a quadriceps snip was conducted to enable better patella tracking of the extensor mechanism and enable a greater degree of implant stability throughout the range of motion. A quadriceps snip was deemed necessary to enable better visualisation and had a minimal effect in improving patella tracking. The authors note that there is no significant morbidity associated with quadricep snips in the literature. It is important to reflect that intraoperative adjustments were based on the opinion of the operating surgeon in balancing the knee and were not strongly correlated to preoperative signs. The patella was not resurfaced due to no significant intraoperative patellofemoral wear. The final implant position allowed satisfactory patella tracking with an equally balanced knee in both flexion and extension with intraoperative knee flexion range achieved up to 90-95 degrees. Closure of the joint included careful repair of the quadriceps and delicate soft tissue handling with absorbable sutures to the skin.

**Figure 2:**
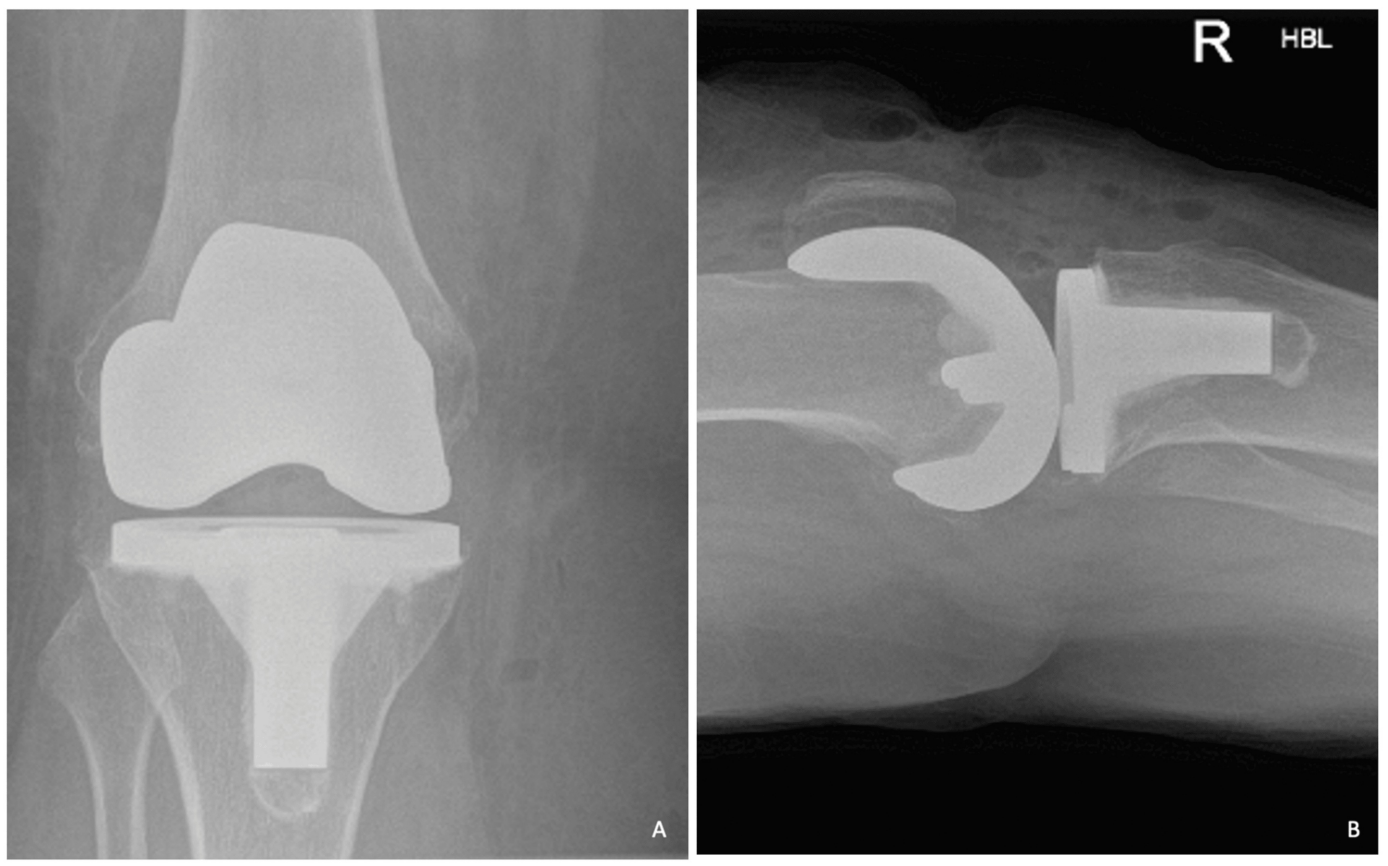
Postoperative radiographic imaging performed immediately after total knee arthroplasty A: Anterior-posterior radiograph of the right knee B: Lateral radiograph of the right knee The figures demonstrate satisfactory implant positioning, lack of intraoperative complications, and restoration of mechanical alignment of the knee joint, which may have contributed to the resolution of the patient's extension deformity.

The patient was not immobilised in any splint but did receive a negative pressure dressing to aid wound healing and ensure any fluid collection inhibiting movement would be prevented. She was placed in a CPM machine for 72 hours with active encouragement from the physiotherapy team. She did not have any intraoperative or postoperative complications with satisfactory postoperative implant check radiographs (Figure 2). The postoperative radiographs demonstrate appropriate positioning of the femoral and tibial components with sufficient external rotation of the components and no concerns for intraoperative features, e.g., fracture. She was discharged once 90 degrees of flexion with the CPM machine was achieved, and a physiotherapy review and satisfactory control of pain were achieved. She was discharged with 90 degrees of active and passive flexion range achieved. Postoperative analgesia consisted of a set protocol initiated by our unit, and the patient's symptoms were well controlled on these medications (paracetamol, ibuprofen, codeine, and opioids as required). 

Follow-up and outcomes

A two-week postoperative telephone review was conducted. The patient reported a marked improvement in her range of motion compared to her preoperative presentation. She reported that at baseline knee flexion was 60 degrees, with improvement to 90-95 degrees on good days. She reported improvement in pain with complete cessation of morphine and use of only simple analgesia (co-codamol and ibuprofen). Furthermore, mobility was stable with no episodes of the knee giving way and with the patient being able to work short distances, with two crutches, comfortably, e.g., around the supermarket and around her home. Furthermore, no postoperative complications were reported, with no concerns for infection, wound dehiscence, or implant-related issues.

At six weeks postoperatively, the patient was reviewed in the clinic. She presented mobilising with one crutch for support. She reported having no pain in the knee and no episodes of instability. She reported only intermittent use of simple analgesia (paracetamol and ibuprofen). Formal pain scores using the visual analogue scale were not quantified, but the patient demonstrated significant improvement subjectively. The range of motion was reported as a maximum flexion range of 65 degrees with full extension and no hyperextension of the limb. She was undergoing vigorous physiotherapy and had a satisfactory record of progress from her physiotherapist. The patient did not report any discomfort going up and down stairs. The reduction in range of motion from the two-week time point was attributed to concerns with compliance with physiotherapy and potential pain inhibition. The wound had healed fully with no concerns for infection or dehiscence. The patient noted an increased size of her knee compared to the contralateral side, and this was explained to be a normal sequelae of knee arthroplasty, which would improve with time. She had not resumed driving but was planning to return to driving in the coming week. The improvement in the patient's ability to mobilise was significant compared to her preoperative wheelchair-bound status, with similar improvements in washing, dressing, and ability to conduct household chores. The patient was overall satisfied with her progress and felt her quality of life, range of motion and mobility had increased greatly due to her treatment.

## Discussion

This case report highlights a rare but life-altering presentation of fixed extension of a native knee. There is no pre-existing literature or instructive guidelines regarding the management of this condition. The lack of previous evidence leads to difficulties in providing a clear, reproducible characteristic profile for this condition. Besides the striking deformity, there is little else to identify in the examination findings. Ligamentous laxity and patella instability were not key concerns in the clinical examination. Furthermore, there were no warning signs in the history heralding the progress/worsening of this condition, and it appeared suddenly and dramatically, according to the patient's detailed account. The presence of patella alta and valgus alignment demonstrated on radiographic imaging would have contributed to the extension deformity; however, the authors do not believe that this was the main causative factor for such a severe presentation as many patients seen in arthroplasty clinic have these radiographic features.

The management that this patient received was the culmination of holistic patient care and joint decision-making. The patient remained aware of the pre-existing risks as well as the possibility of the knee remaining locked in extension post-procedure. However, successful surgical management demonstrates the life-changing effect that correct treatment can have on a patient [6]. The use of arthroplasty was essential in this case, and the role of progressive soft tissue releases and addressing the degenerative joint disease was enough to enable a functional flexion range of motion. The patient was very satisfied with her outcome at the six-week mark, and the authors are confident that she will continue to make progress. The effect on activities of daily living is marked by the transformation of a wheelchair-bound patient to a person who can re-engage with society, conduct self-care, and have the confidence to pursue work in the future. The effect of arthroplasty on pain management is also notable, with decreased opioid requirements at the six-week follow-up likely being attributed to greater confidence in mobilisation, reduction in pain sensitivity, and treatment of the degenerative joint disease (and the pain associated with arthritis).

From the perspective of causation, it is difficult to stratify an exact cause for this patient's presentation. Certainly, there is likely to be a biopsychosocial element to her condition. The formation of scar tissue with degeneration of her meniscus on the background of osteoarthritis combined with elements of quadriceps inhibition and anxiety may have contributed to her knee extension. Certainly, the presence of severe scalloping over the lateral tibial plateau may have provided both a mechanical and non-mechanical cause of her symptoms, as she may have been apprehensive about rolling her femur into the tibia. Hence, in such a complex situation, joint decision-making and sequential surgical investigation are imperative. The initial use of arthroscopy and manipulation under anaesthesia was a useful diagnostic and therapeutic tool to identify and differentiate between mechanical versus non-mechanical causes of her deformity [7]. Furthermore, the decision to pursue total joint arthroplasty enables range of motion to be restored. It should be noted that the full range of motion was not restored in this case; however, the gained range of motion was acceptable to the patient and means that she can conduct her activities of daily living without concern.

The limitations of this study include the limited duration of follow-up which is acknowledged by the authors. The patient will remain under the care of the operating team and planned non-urgent follow-up will be initiated at the six-month and one-year time points post-surgery. The authors also acknowledge the requirement of patient-reported outcome measures (PROMS) scoring/pain scoring, which would have helped stratify objective improvement in the patient's functional outcomes and pain score. Furthermore, this is a single, isolated cause of a knee locked in full extension; future studies should focus on a large case series to draw stronger conclusions regarding optimal management strategies.

## Conclusions

This case report highlights a rare case of a native arthritic knee locked in extension. Total knee arthroplasty was demonstrated to be a suitable management choice with positive, life-altering postoperative outcomes for the patient. Future studies should focus on a larger case series to provide stronger evidence for the use of total joint arthroplasty in such deformity cases.
